# Supplementary material for: AMD-FV: Adaptive margin loss and dual path network+ for deep face verification
Source: PLoS One. 2025 May 28;20(5):e0324485. doi: 10.1371/journal.pone.0324485 (PMC12118897; doi:10.1371/journal.pone.0324485)
Supplement: S1 Appendix — Magnitude of Cross Product. (PDF) [file pone.0324485.s001.pdf]

Appendix

A. Magnitude of Cross Product

This section provides the mathematical details for the derivation of the magnitude of the cross product, which contributes to the margin hyperparameter m of the Adaptive Margin Loss in Eq (**??**).

Given the trigonometric function, we substitute $\cos\theta_j$ and $\sin\theta_j$ from the equations of the dot product ($W_j^{(T)} x_i$) and the magnitude of the cross product ($|W_j^{(T)} \times x_i|$). Then we simplify the equation for the magnitude of the cross product. We adhere to the assumption of the CosFace [1] and the ArcFace [2] model, i.e., they normalized features x_i, and weights W_j. The magnitude of the cross product is formulated as follows:

$$W_j^{(T)} x_i = \|W_j\| \|x_i\| \cos\theta_j$$
$$|W_j^{(T)} \times x_i| = \|W_j\| \|x_i\| \sin\theta_j$$

Extracting the values of $\cos\theta_j$ and $\sin\theta_j$ for trigonometric function as:

$$\cos^2\theta_j + \sin^2\theta_j = 1$$
$$\cos\theta_j = \frac{W_j^{(T)} x_i}{\|W_j\| \|x_i\|}$$
$$\sin\theta_j = \frac{|W_j^{(T)} \times x_i|}{\|W_j\| \|x_i\|}$$
$$\left(\frac{W_j^{(T)} x_i}{\|W_j\| \|x_i\|} \right)^2 + \left(\frac{|W_j^{(T)} \times x_i|}{\|W_j\| \|x_i\|} \right)^2 = 1$$

Following the assumption of the CosFace [1] and the ArcFace [2] model, i.e., the norm of features x_i, and norm of weights W_j, as 1 ($\|x_i\| = \|W_j\| = 1$).

$$\left(W_j^{(T)} x_i \right)^2 + \left(|W_j^{(T)} \times x_i| \right)^2 = 1$$
$$\left(|W_j^{(T)} \times x_i| \right)^2 = 1 - \left(W_j^{(T)} x_i \right)^2$$

$$|W_j^{(T)} \times x_i| = \sqrt{1 - \left(W_j^{(T)} x_i \right)^2} \quad (1)$$

B. Range of AML

This section provides the mathematical details for the derivation of range for the Adaptive Margin Loss, which contributes to the scale hyperparameter s of the AML in Eq (**??**).

Given that AML follows the CosFace [1] and the ArcFace [2] assumption, i.e., norm of features x_i and norm of weights W_j, as 1 ($\|W_j\|= 1$, $\|x_i\|= 1$); then the activation of the last FC layer ($W_j^{(T)} x_i = \|W_j\| \|x_i\| \cos\theta_j$) depends on $\cos\theta_j$, which depicts the similarity. Whereas, we subtract the dissimilarity estimate m (Eq (**??**)) from the similarity estimate $\cos\theta_j$ (Eq (**??**)). In fact, we are creating a function f_j by subtracting the magnitude of the cross product ($|W_j^{(T)} \times x_i|$) from dot product ($W_j^{(T)} x_i$) as follows:

$$
\begin{gathered}
f_j = \left(W_j^{(T)} x_i\right) \quad - \quad \left(|W_j^{(T)} \times x_i|\right) \\
where \\
W_j^{(T)} x_i = \|W_j\| \|x_i\| \cos\theta_j \\
|W_j^{(T)} \times x_i| = \|W_j\| \|x_i\| \sin\theta_j \\
As \quad \|W_j\|= 1, \|x_i\|= 1 \\
f_j = \cos\theta_j - \sin\theta_j \\
\sin\theta_j \quad is \quad just \quad a \quad phase-shift \quad of \quad \cos\theta_j \\
\cos\theta_j = \sin(\theta_j + \pi/2)
\end{gathered}
$$

$$so \quad f_j = \sin(\theta_j + \pi/2) - \sin\theta_j \tag{2}$$

The resultant sin function is expressed as the addition of two or more sin functions, with different multiples, possibly by different phase shifts. When a constant is multiplied by the sin function, the amplitude of the sin function increases or decreases (depending on positive or negative values), therby determining the range of the *sin* function. Therefore, we transformed the resultant into an equation such that the Law of Cosines [3] can be applied and the amplitude of the function can be calculated. Thus, the proper formulation of function $\sin(\theta_j + \pi/2) - \sin\theta_j$ with constants becomes:

$$\sin(\theta_j + \pi/2) - \sin\theta_j \tag{3}$$

$$= \ (1)\sin(\theta_j + \pi/2) + (-1)\sin(\theta_j + 0) \tag{4}$$

Law of cosines [3] implies:

$$c\sin(\theta_j + C) = a\sin(\theta_j + A) + b\sin(\theta_j + B) \tag{5}$$

where A, B, C, a, b and c are constants, and calculating the constant c, leads us to the range of the function. Through the law of cosines, using the Eq (3) and

Eq (5), we calculate the value for the amplitude c, as follows:

$$c = \sqrt{a^2 + b^2 + 2ab\cos(B - A)}$$
$$As \quad a = 1, b = -1, A = \pi/2, B = 0$$
$$c = \sqrt{(1)^2 + (-1)^2 + 2(1)(-1)\cos(0 - \pi/2)}$$
$$c = \sqrt{1 + 1 - 2\cos(-\pi/2)}$$
$$c = \sqrt{2}$$

$$Range = [-\sqrt{2}, \sqrt{2}] \tag{6}$$

AML deals with the difference; therefore, we consider the positive value of the range i.e., $\sqrt{2}$ to be R.

References

[1] Wang H, Wang Y, Zhou Z, Ji X, Gong D, Zhou J, et al. CosFace: Large Margin Cosine Loss for Deep Face Recognition; 2018. p. 5265–5274. Available from: http://arxiv.org/abs/1801.09414 https://ieeexplore.ieee.org/document/8578650/.

[2] Deng J, Guo J, Xue N, Zafeiriou S. ArcFace: Additive Angular Margin Loss for Deep Face Recognition; 2019. p. 4690–4699. Available from: http://arxiv.org/abs/1801.07698.

[3] Pickover C. The math book: from Pythagoras to the 57th dimension, 250 milestones in the history of mathematics; 2009. Available from: http://choicereviews.org/review/10.5860/CHOICE.47-3221.
