# Supplementary material for: AMD-FV: Adaptive margin loss and dual path network+ for deep face verification
Source: PLoS One. 2025 May 28;20(5):e0324485. doi: 10.1371/journal.pone.0324485 (PMC12118897; doi:10.1371/journal.pone.0324485)
Supplement: Attachment — Submitted filename: PONE-D-24-28461R1- Response to the Reviewers comments.pdf [file pone.0324485.s002.pdf]

Reviewer #1:

We would like to thank the reviewer for their rigorous review of our work and their constructive comments, which have led to substantial improvement in the presentation and communication of the theory and results presented in this work. In what follows

R1.C1- The abstract lacks an explanation of the proposed algorithm in simple and clear steps, in addition to not stating the type of metrics and their values used to demonstrate the efficiency of the proposed algorithm.

Response: Thank you for your helpful comment. The abstract has been re-written to provide a clear explanation of the proposed approach, including the results of the metrics used to demonstrate performance:

"Face verification is important in a variety of applications, for instance, access control, surveillance, and identification. Existing methods often struggle with the challenges of dataset imbalance and manual tuning of hyperparameters. To address this, we propose the Adaptive Margin Loss and Dual Path Network+ (AMD-FV) for deep face verification. Two innovations are introduced, namely, Adaptive Margin Loss (AML) and Dual Path Network+ (DPN+). AML at automating the selection of margin and scale hyperparameters in large margin loss functions, thus, eliminating the need for manual tuning. Input dissimilarity information is used to estimate the margin, while the scale parameter is computed using the number of classes and AML's range. Next, DPN+ enhances the original Dual Path Network by redesigning the first block with a series of 3x3 convolutions, batch normalization, and ReLU activations, leveraging shared connections across layers, leading to increases in spatial resolution and computational cost efficiency, while maximizing the use of discriminative features. We present comprehensive experiments on five diverse face verification datasets (LFW, Megaface, IJB-B, CALFW, and CPLFW) to demonstrate the effectiveness of the proposed approach. The results show that AMD-FV outperforms state-of-the-art methods, achieving a verification accuracy of 99.75% on LFW, improving the True Acceptance Rate by 6% on IJB-B at a False Acceptance Rate of 0.001, compared to VGGFace2, and attaining a Rank-1 identification score of 92.16% on Megaface, surpassing the CosFace model by 9.44%."

R1.C2- No keywords found.

Response: Thank you for your suggestion. The PLOS One journal template does not explicitly incorporate keywords section, which is the reason why keywords were not identified in the original submission. To accommodate the reviewer's comment, a new section "Keywords" was added, immediately after the abstract, to capture the key technical terms involved in the proposed work:

"Keywords

Adaptive Margin Loss, Face verification, Deep learning, Dual Path Network+"

R1.C3- The abbreviation is added to the scientific name only at the first appearance and not at every appearance, for example Adaptive Margin Loss (AML).

Response: We apologize for the oversight. The manuscript has been thoroughly revised to ensure that abbreviations are only defined at first appearance.

R1.C4- Ensure that the research papers included in the Related Works section are arranged

according to the year of publication (from oldest to newest). In addition to stating the negatives found in all or some of these papers.

Response: Thank you for the helpful suggestion. References have been arranged in chronological order, and a detailed critique of the works has been provided in the revised manuscript:

"Margin-based loss functions, such as SphereFace [8], CosFace [9], and ArcFace [10], demonstrated significant success in face verification tasks. The hyperparameters associated with these loss functions, often manually chosen, play a crucial role on the performance of the deep learning model. Indeed, suboptimal or incorrect selection of these hyperparameters can significantly affect their properties and, subsequently, model performance. Typically, determining these parameters involves a time-consuming and laborious trial-and-error process, the success of which is limited by the choice of initial parameters. To address this issue, researchers have proposed data-driven methods to estimate these hyperparameters. For instance, Liu et al., [11] argue that face data typically consists of large-scale classes and samples, emphasizing that selecting appropriate classes and sample numbers for training helps the model achieve better generalization. They proposed the use of hard prototype mining and introduced the AdaptiveFace model to estimate the associated hyperparameters. However, the computational overhead of learning individual margins for each class thoroughly considered. In the case of large-scale datasets, this could potentially lead to significant increases in training time and memory requirements. Moreover, as demonstrated in the experiments, the performance of the method is sensitive to the choice of the hyperparameter controlling the strength of the margin constraint. Zhang et al., [12] propose the Adacos model to tackle the challenge of hyperparameter selection in face verification tasks. However, their approach presents a number of challenges. First, it assumed that during training, the sum of logits for non-corresponding classes remains almost constant. Beyond empirical evidence, the theoretical justification for this assumption, crucial in deriving the adaptive scale parameter, is not proven. Moreover, the analysis of probability curves is based on simplified models, which may not fully capture the complexity of real-world face recognition scenarios, e.g., class imbalance and samples of varying difficulty.

Zhang et al., [25], propose diversity regularization by training the margin hyperparameter and constraining it within the range of 0.5 and π. While the authors automatically train the margin hyperparameter, they introduce two additional manual parameters in terms of the range. Moreover, there is insufficient analysis of the impact of the balance hyperparameter across different datasets and network architectures, and the computational overhead associated with the calculation of adaptive margins and diversity regularization. RamFace [26] addresses racial bias by proposing an adaptive margin loss based on intra-identity compactness using multitask learning. Two loss functions, i.e., race adaptive margin-based loss and race classification loss, are introduced, however, the additional hyperparameter λ is used to balance them. There is insufficient consideration on the appropriate choice of this hyperparameter, and its impact on model performance. In addition, the manuscript neither provides formal guarantees on the fairness properties of the proposed method, nor does it discuss in sufficient detail the potential trade-offs between overall accuracy and fairness across different racial groups. EnhanceFace [27], proposes adaptive weighted softmax loss by segregating hard samples into semi-hard and harder samples. The loss function adjusts the corresponding weights during training. The authors introduce the enhance rate ω, used to adjust the effects of semi-hard samples. However, the selection of this parameter appears arbitrary, as there is lack of justification for the chosen range and the step size. JAMsFace [28] proposes a joint adaptive margin loss function, which learns class-related margins by incorporating margin penalties within the cosine angle of a loss function.

These models mitigate the problem to some extent. However, they only provide a means of estimating a single hyperparameter, i.e., margin, and moreover, there is a lack of a clear formulation on how this is calculated based on class distribution. In contrast, the proposed AML approach estimates both the scale and margin hyperparameters.

In the context of CNN architectures, García et al., [21] discussed the difficulty of preserving diagnostic features in medical images during downsampling. He et al., [29] introduced the Residual Network (ResNet), which utilizes skip connections to enable very deep networks, addressing optimization challenges. However, the use of downsampling in ResNet leads to significant information loss. Sedlar et al., [22] identified similar challenges in genomic signal processing, calling for strategies that balance data reduction with feature retention. Zhao et al., [23] proposed random shifting to minimize systematic information loss during downsampling. In contrast, Huang et al., [30] introduced the Dense Convolutional Network (DenseNet), which uses skip connections to concatenate features from earlier layers, but at the expense of high computational complexity. Chen et al., [20] demonstrated that ResNet reuses features via residual paths, while DenseNet explores new features through densely connected paths. Building on this, Chen et al., [20] proposed the Dual Path Network (DPN), which combines the strengths of both ResNet and DenseNet, although it still suffers from information loss due to its downsampling approach. The proposed Dual Path Network+ is an enhancement to the original DPN, improving spatial resolution and computational efficiency, while leveraging shared connections across layers to maximize feature utilization."

R1.C5- The comparison presented by the researcher between the submitted work and other works needs more clarification and expansion in the discussion of the included tables and charts.

Response: We are sincerely grateful to the reviewer for their recommendation. To address this comment, we substantially expanded the Results section, by including a comprehensive ablation study section to provide a detailed and comprehensive discussion of the results. This section highlights the improvements introduced by each of the components in the proposed AMD-FV framework, with a clear explanation of how the performance metrics compare to those in state-of-the-art works, as follows:

"Ablation study

The ablation study provides evidence in support of the justification for the key components of AMD-FV. Five models are evaluated, using the architecture of a 68 layered DPN+ neural network. Their summary descriptions are shown in Table 3.

The models were analyzed according to several performance metrics, such as model complexity (i.e., parameter number and model size), computational time measured in FLOPs, inference time in seconds, and accuracy rate. Note that model complexity, computational time, and inference time are closely related to the computational power of the machine, whereas accuracy rate is related to model performance on the input data. Tables 4 and 5 illustrate the computational complexity and inference times of the evaluated models, respectively. It should be noted that the same CNN architecture was used in all of the models. Therefore, the chosen metrics will have similar values, providing the means to perform a fair comparison of impact of loss functions on the basis of the accuracy rate.

Robustness of DPN+

In this sub-section, we focus on the analysis of the proposed Dual Path Network+ over the Dual Path Network, so as to provide a fair comparison. Both DPN and DPN+ were trained on the same dataset (MS1Mv2 [10]), under the same settings (See Section for details) and loss function (i.e., CosFace loss [9]). The models were then evaluated on the five benchmark face datasets. The obtained results support the claim that the proposed DPN+ model (DPN+68-C) extracts more discriminative features than those of the DPN model (DPN68-C). The verification accuracy of DPN+68-D is 0.17%, 1.78% and 2.94% higher than that of DPN68-C on the LFW, CPLFW and Megaface datasets, respectively (Table 9). A slight drop of 0.07% in the performance of the DPN+68-C compared to DPN68-C is observed in the case of the CALFW dataset. Finally, the ROC curves in Fig. 3 demonstrate that the DPN+68-C model significantly surpasses the DPN68-C model on the IJB-B dataset.

Robustness of AMD-FV

Next, we provide a comparison between the most relevant state-of-the-art angular loss function models, and the proposed AMD-FV model. The SphereFace model [8] proposes a multiplicative angular margin, where the margin value is multiplied by the angle, which, in turn, provides for a fixed distance between the samples. The multiplicative property of the margin highly influences the angle. When the angle decreases towards zero, the margin vanishes (see Fig. 6(a)). The CosFace [9] and ArcFace models [10] introduce the additive angular margin. In CosFace model [9], the margin value is subtracted from the cosine value (Eq. 2), which overcomes the limitation of the vanishing margin (see Fig. 6(b)). Compared with the CosFace model [9], the ArcFace model [10] adds a margin to the angle. The ArcFace model [10] also has a vanishing margin problem. However, because of the additive property, a fixed margin value does not significantly affect the angle (see Fig. 6(c)). In the proposed model, AML automatically estimates the margin using the input data, so that it does not vanish. Specifically, it uses the cross product of the feature map to estimate the margin according to Eq. 3 and subtracts the margin from the cosine value (Eq. 5) (see Fig. 6(d)). By adaptive estimation of the margin and the scale hyperparameters, AML provides an alternative to the otherwise, time-consuming training of the trial and error approach.

The principal objective in this research is the development of an automated procedure for the selection of the margin and scale hyperparameters, which resulted to the proposed AML function. Analysis of the experimental results demonstrates that the accuracy obtained by the AMD-FV model surpasses the performance of both state-of-the-art AdaptiveFace [11] and Adacos models [12]. Note that the mean verification accuracy on the LFW dataset is almost saturated and thus, a small increase in the accuracy has a significant impact. The verification accuracy achieved by the AMD-FV model is 99.75%, which is higher than that of AdaptiveFace at 99.63% and Adacos at 99.71% (Table 6). In addition, the accuracy of the AMD-FV model is higher than that of the baseline model (DPN+68-A) on all the testing datasets. It is worth noting that the introduction of AML results to an average improvement of 2.338% compared to using CESL (Table 10 and Fig. 4). The verification accuracies obtained by the DPN+68-B model (DPN+ with ArcFace loss) and the DPN+68-D model are similar to one another (Table 8). Specifically, the use of ArcFace loss led to outperforing the DPN+68-D model by a margin of 0.19%, on average, suggesting that the proposed adaptive DPN+68-D model not only surpasses the state-of-the-art adaptive margin-based models, but also favorably compares with the manually optimized margin-based model (i.e., ArcFace). Fig. 5 provides a visual comparison of the computational complexity of the DPN+68-D model and the ArcFace model [10] (i.e., ResNet with 100 layers). It is shown that the proposed AMD-FV model leads to substantial reductions of 32%, 26.92% and 41.91% in terms of the number of layers, parameters and FLOPS, respectively.

Comparison with state-of-the-art methods

In this sub-section, we consider the performance of AMD-FV in regards to related state-of-the-art approaches. The mean verification accuracies acquired on the LFW dataset are presented in Table 11. The proposed AMD-FV method attains a competitive mean verification accuracy of 99.83%, surpassing the performance of the majority of previously published methods e.g., [42], [8], [43], and [44]. Arcface [9] exhibits a slightly higher accuracy compared to AMD-FV by 0.08%. However, in comparison to the manually optimized CosFace model [9], the proposed AMD-FV method with automated hyperparameter selection demonstrates an improvement of 0.02%. This suggests that the automated hyperparameter selection process of AMD-FV could serve as an alternative to the empirically determined hyperparameter values of the CosFace model, eliminating the need for time-consuming trial and error. Furthermore, in comparison to the other two adaptive models, namely AdaptiveFace [11] and Adacos [12], AMD-FV demonstrates a marginal improvement of 0.13% and 0.04%, respectively.

IJB-B is considered to be a challenging dataset concerning occlusion, poses, and illumination. In Table 12, the True Acceptance Rate (TAR) is presented concerning various False Acceptance Rates (FAR) on the IJB-B dataset. The proposed AMD-FV model outperforms all other models. Specifically, it improves on the VGGFace2 model by 6%, and by 4.6% and 3.9% compared to MN-V and MN-VC, respectively, at a FAR of 0.001. At a FAR of 0.01, the proposed AMD-FV surpasses VGGFace2, MN-V, and MN-VC models by 2.83%, 2.23%, and 1.93%, respectively. At a FAR of 0.1, AMD-FV's performance marginally outperforms that of the VGGFace2, MN-V, and MN-VC. This insight underscores the improved performance of AMD-FV compared to state-of-the-art methods on the IJB-B dataset.

The Rank-1 identification scores of existing methods on the Megaface dataset are presented in Table 13. The gallery set of 1M distractor images in Megaface presents a wide range of poses, ages, and illumination changes, making this dataset exceptionally challenging for face verification. Notably, the proposed AMD-FV method outperforms several state-of-the-art models, such as the CosFace model [9], Sphereface model [8], and FaceNet [42], with considerable margins (i.e., greater than 10%). However, when compared to the adaptive hyperparameter estimation models of AdaptiveFace [11] and Adacos [12], the results of AMD-FV are lower. This difference is attributed to Megaface's use of a large number of distractor images in its verification protocol, which affects AMD-FV's margin hyperparameter estimation values computed based on the mean. It is worth noting that the primary objective of this work is to adaptively estimate the large margin cosine loss of CosFace [9]. In this regard, the proposed AMD-FV model achieves a Rank-1 score of 92.16%, significantly surpassing CosFace by 9.44%.

The mean verification accuracy on the Cross Age LFW (CALFW) [39] and Cross Pose LFW (CPLFW) datasets [40] is presented in Table 14. These datasets extend the LFW dataset by introducing variations in age and pose, thereby increasing the complexity level. On the CALFW dataset, AMD-FV significantly outperforms the Center loss [47], SphereFace [8], and VGGFace2 models [5] by 10%, 5.21%, and 4.94%, respectively. Similarly, on the CPLFW dataset, the mean verification accuracy of AMD-FV surpasses that of the Center loss [47], SphereFace [8], and VGGFace2 models [5] by 13.52%, 9.60%, and 7.00%, respectively."

Moreover, a number of new Tables and Figures were included to support these additional results and discussions. In the revised manuscript, the following new Tables were included:

Table 3: overview of the experimental models used in the ablation study

Table 4: performance comparison of the models w.r.t computational parameters

Table 5: performance comparison w.r.t inference times

Table 7: comparison of mean verification rates between AMD-FV and ArcFace loss

Table 8: comparison of MVA, precision, and recall with ArcFace loss

Table 9: comparison between DPN and DPN+ using CosFace loss

Table 10: comparison bebetween AMD-FV and baseline model

Moreover, the following new Figures were included:

Figure 3: ROC comparison between DPN and DPN+ using CosFace loss

Figure 4: ROC comparison between AMD-FV and baseline model

Figure 5: Computational parameters comparison between ArcFace and AMD-FV

Figure 6: Geometric representation of angular loss functions

R1.C6-No numerical values were included for the results in the conclusions addressed by the researcher.

Response: Thank you for this valuable suggestion. We expanded the conclusions sections, by including an overview of the results and improvements due to AMD-FV. Moreover, we expanded the future work as follows:

"*...Overall, the AMD-FV model demonstrates competitive performance with the advantages of automated hyperparameter selection. Specifically, the proposed framework achieves verification accuracy of 99.75% on the LFW dataset, outperforming AdaptiveFace (99.62%) and Adacos (99.71%). Moreover, it delivers TAR of 94.8%, 97.3% and 98.9% at FAR of 0.001, 0.01 and 0.1, respectively, on the IJB-B dataset, surpassing VGGFace2 by up to 6%. Similarly, competitive performances are delivered on Megaface with Rank-1 identification scores of 92.16%, compared to CosFace, and MVA of 95.51% and 91%, respectively, on CALFW and CPLFW. In regards to computational efficiency, AMD-FV achieves significant reductions in terms of the number of layers (32%), system parameters (26.92%) and FLOPs (41.91%), compared to ArcFace, positioning it as an attractive solution to real-world applications. A number of potential directions will be pursued as part of further research, including cross-domain applications, e.g., expand the use of AMD-FV in person re-identification, iris recognition, and fingerprint recognition, thus evaluating its adaptability. Moreover, we intend to combine the proposed system with other biometric modalities, for instance, gait, and consider its authentication accuracy in complex scenarios. Further research will evaluate its resilience against adversarial attacks to ensure security in such critical applications.*"

R1.C7- There are no published references in 2024, so it is preferable to include at least two research papers published in the above-mentioned year.

Response: Thank you for the helpful suggestion. In response to this, the introduction section was updated to include three recently published papers from 2024 to strengthen the manuscript's relevance and contemporality. The first paper, HAMFace: Hardness Adaptive Margin Loss for Face

Recognition with Various Intra-class Variations (2024) [13], introduces a Hardness Adaptive Margin Loss that dynamically adjusts hyperparameters for improved intra-class distribution, particularly addressing challenges posed by hard positive samples. The second paper, Regularization Using Noise Samples Identified by the Feature Norm for Face Recognition (2024) [14], proposes a novel regularization approach utilizing the L2 norm of features and Noise Direction Regularization to enhance face recognition performance. Finally, CoReFace: Sample-guided Contrastive Regularization for Deep Face Recognition (2024) [15] presents an adaptive margin-based contrastive loss method with enhancements to the scale and margin hyperparameters for superior feature learning. These additions provide a robust foundation and contextual relevance for the proposed work, as follows:

"*For example, HAMFace [13] introduces a Hardness Adaptive Margin Loss, applying larger margins to challenging positive samples and using a control hyperparameter to jointly manage the margin and scale hyperparameters. The study by Gim et al., [14] incorporates Noise Direction Regularization (NDR), leveraging the L2 norm of the features as a regularizer and introducing a hyperparameter to improve performance. CoReFace [15] further advances the field by employing an adaptive margin-based contrastive loss, which utilizes an exponential moving average for the margin, while relying on manual scale tuning.*"

Reviewer #2:

This manuscript presents a Face verification based on Adaptive Margin Loss and Dual Path Network+. Although there are extensive experiments, the author did not clarify the appropriate motivations and limitations.

Response: We would like to thank the reviewer for identifying the positive aspects of our work, and for their helpful suggestions, which led to improving the quality and presentation of the manuscript.

Please address the following comments for your next revisions:

R2.C1- The fourth contribution is not really a contribution. This is a standard procedure for verification of the performance of the proposed method.

Response: Thank you for your suggestion. We removed the 4th contribution from the corresponding list, and strengthened the clarity of the remaining contributions.

R2.C2- The motivation seems weak, please try to focus on other methods limitations and try to optimize your work accordingly.

Response: We sincerely appreciate this helpful recommendation. To address this, we provided a thorough critique of the limitations of other works, and optimized the flow of the work as follows:

"*From a loss function design perspective, SphereFace [8] introduces a multiplicative angular margin loss function. CosFace [9] demonstrates the effectiveness of subtracting a margin rather than multiplying it. ArcFace [10] proposes an additive margin approach, which facilitates the learning of discriminative features. However, the limitation of SphereFace [8] is that its performance is sensitive to hyperparameters, particularly the choice of the angular margin parameter. Indeed, improper tuning of this parameter may lead to suboptimal feature discrimination. CosFace [9] exhibits computational complexity, especially when dealing with large-scale datasets. Specifically, the computation of the cosine similarity with respect to each class centroid during training can become computationally expensive, potentially affecting training times and resource efficiency. ArcFace [10] relies on hyperparameters that necessitate manual selection, involving repeated trial and error, which is time-consuming. Thus, on one hand, the hyperparameters of angular margin loss functions offer leverage for tuning deep learning architectures to achieve improved results. On the other hand, improper selection of these hyperparameters may substantially impact accuracy.*

To the best of our knowledge, to date, AdaptiveFace [11] and AdaCos [12] are the primary methods offering automated hyperparameter selection for large margin loss frameworks. AdaptiveFace [11] estimates the margin hyperparameter, while AdaCos [12] focuses on the scale hyperparameter. Several related approaches address various aspects of hyperparameter optimization. For example, HAMFace [13] introduces a Hardness Adaptive Margin Loss, applying larger margins to challenging positive samples and using a control hyperparameter to jointly manage the margin and scale hyperparameters. The study by Gim et al., [14] incorporates Noise Direction Regularization (NDR), leveraging the L2 norm of the features as a regularizer and introducing a hyperparameter to improve performance. CoReFace [15] further advances the field by employing an adaptive margin-based contrastive loss, which utilizes an exponential moving average for the margin, while relying on manual scale tuning. To overcome the limitations of

existing methods, this study proposes AML, a novel framework that automates the selection of both margin and scale hyperparameters. This estimates the margin using input dissimilarity information derived from the cross-product of the feature and weight vectors, while the scale hyperparameter is determined based on the number of classes and the effective range of AML (more details in Adaptive Margin Loss).

From a network architecture design perspective, the Deep Residual Network (ResNet) [16] introduced skip connections, where each micro-block (residual function) includes a residual path. This path element-wise adds input features to the output of the same micro-block, defining it as a residual unit. ResNet evolved into various architectures such as Inception-Resnet [17] and ResNeXt [18], each characterized by changes in their structural designs. Later, [19] proposed the Dense Convolutional Network (DenseNet), which uses dense connections, with each layer receiving inputs from all preceding layers and passing its own feature maps to all subsequent layers, thus enhancing information flow and feature reuse. Chen et al., [20] explore the relationship between residual networks and densely connected networks, demonstrating that residual networks exhibit characteristics of densely connected networks when connections are shared across layers. They proposed the Dual Path Network (DPN), which leverages the residual path for implicit feature reuse and continuous exploration of new features through the densely connected path.

Several studies [16–20] rely on downsampling in the network architecture, however, this often leads to significant information loss. Garcia et al., [21] highlighted the challenges of preserving diagnostic features in medical images, proposing feature-preserving techniques, which, despite improvements, were unable to completely prevent degradation. Similarly, Sedlar et al., [22] noted that inappropriate downsampling in genomic signal processing obscured critical patterns, advocating for tailored rules to optimize the balance between data reduction and feature retention. Zhao et al., [23] introduced random shifting to minimize systematic information loss in CNN downsampling layers, enhancing feature detail preservation. Kuen et al., [24] proposed stochastic downsampling to improve network regularization and enable cost-adjustable inference, however, this introduces variability, which could affect feature consistency. To address this limitation, we introduce the Dual Path Network+ with a carefully redesigned Block-1, which includes a series of 3x3 convolutions, batch normalization, and ReLU activation functions to enhance spatial resolution and reduce computational costs. Moreover, DPN+ maintains shared connections across layers to maximize the utilization of discriminative features, thereby enhancing both performance and efficacy. More details can be found in Dual Path Network+."

Moreover, we explicitly stated the motivation for this research as follows:

"*In summary, existing approaches suffer due to the requirement for manual hyperparameter selection, which significantly impacts on their performance, or alternatively, facing computational challenges, when it comes to dealing with large-scale, real-world datasets. Moreover, due to the use of downsampling in the CNN architectures, there is significant information loss, reducing the discriminating ability of the underlying features.*"

R2.C3- The related work section is limited to few works, and there are no expansion to other pipelines, this should be reflected on your work motivations. Try to reorganize your related work section to mathematical models, ML and DL models.

Response: The related work section has been expanded to accommodate the reviewer's comment. Moreover, the work has been reorganized (e.g., loss-based and DL-based) and focused

on identifying potential weaknesses in the state of the art, further reinforcing the motivation for the current work:

"Margin-based loss functions, such as SphereFace [8], CosFace [9], and ArcFace [10], demonstrated significant success in face verification tasks. The hyperparameters associated with these loss functions, often manually chosen, play a crucial role on the performance of the deep learning model. Indeed, suboptimal or incorrect selection of these hyperparameters can significantly affect their properties and, subsequently, model performance. Typically, determining these parameters involves a time-consuming and laborious trial-and-error process, the success of which is limited by the choice of initial parameters. To address this issue, researchers have proposed data-driven methods to estimate these hyperparameters. For instance, Liu et al., [11] argue that face data typically consists of large-scale classes and samples, emphasizing that selecting appropriate classes and sample numbers for training helps the model achieve better generalization. They proposed the use of hard prototype mining and introduced the AdaptiveFace model to estimate the associated hyperparameters. However, the computational overhead of learning individual margins for each class thoroughly considered. In the case of large-scale datasets, this could potentially lead to significant increases in training time and memory requirements. Moreover, as demonstrated in the experiments, the performance of the method is sensitive to the choice of the hyperparameter controlling the strength of the margin constraint. Zhang et al., [12] propose the Adacos model to tackle the challenge of hyperparameter selection in face verification tasks. However, their approach presents a number of challenges. First, it assumed that during training, the sum of logits for non-corresponding classes remains almost constant. Beyond empirical evidence, the theoretical justification for this assumption, crucial in deriving the adaptive scale parameter, is not proven. Moreover, the analysis of probability curves is based on simplified models, which may not fully capture the complexity of real-world face recognition scenarios, e.g., class imbalance and samples of varying difficulty.

Zhang et al., [25], propose diversity regularization by training the margin hyperparameter and constraining it within the range of 0.5 and π. While the authors automatically train the margin hyperparameter, they introduce two additional manual parameters in terms of the range. Moreover, there is insufficient analysis of the impact of the balance hyperparameter across different datasets and network architectures, and the computational overhead associated with the calculation of adaptive margins and diversity regularization. RamFace [26] addresses racial bias by proposing an adaptive margin loss based on intra-identity compactness using multitask learning. Two loss functions, i.e., race adaptive margin-based loss and race classification loss, are introduced, however, the additional hyperparameter λ is used to balance them. There is insufficient consideration on the appropriate choice of this hyperparameter, and its impact on model performance. In addition, the manuscript neither provides formal guarantees on the fairness properties of the proposed method, nor does it discuss in sufficient detail the potential trade-offs between overall accuracy and fairness across different racial groups. EnhanceFace [27], proposes adaptive weighted softmax loss by segregating hard samples into semi-hard and harder samples. The loss function adjusts the corresponding weights during training. The authors introduce the enhance rate ω, used to adjust the effects of semi-hard samples. However, the selection of this parameter appears arbitrary, as there is lack of justification for the chosen range and the step size. JAMsFace [28] proposes a joint adaptive margin loss function, which learns class-related margins by incorporating margin penalties within the cosine angle of a loss function. These models mitigate the problem to some extent. However, they only provide a means of estimating a single hyperparameter, i.e., margin, and moreover, there is a lack of a clear

formulation on how this is calculated based on class distribution. In contrast, the proposed AML approach estimates both the scale and margin hyperparameters.

In the context of CNN architectures, García et al., [21] discussed the difficulty of preserving diagnostic features in medical images during downsampling. He et al., [29] introduced the Residual Network (ResNet), which utilizes skip connections to enable very deep networks, addressing optimization challenges. However, the use of downsampling in ResNet leads to significant information loss. Sedlar et al., [22] identified similar challenges in genomic signal processing, calling for strategies that balance data reduction with feature retention. Zhao et al., [23] proposed random shifting to minimize systematic information loss during downsampling. In contrast, Huang et al., [30] introduced the Dense Convolutional Network (DenseNet), which uses skip connections to concatenate features from earlier layers, but at the expense of high computational complexity. Chen et al., [20] demonstrated that ResNet reuses features via residual paths, while DenseNet explores new features through densely connected paths. Building on this, Chen et al., [20] proposed the Dual Path Network (DPN), which combines the strengths of both ResNet and DenseNet, although it still suffers from information loss due to its downsampling approach. The proposed Dual Path Network+ is an enhancement to the original DPN, improving spatial resolution and computational efficiency, while leveraging shared connections across layers to maximize feature utilization."

R2.C4- There is no proof of concept in each step in your work.

Response: We are sincerely grateful to the reviewer for their recommendation. To address this comment, we substantially expanded the Results section, by including a comprehensive ablation study section to provide a detailed and comprehensive discussion of the results. This section highlights the improvements introduced by each of the components in the AMD-FV framework, providing justification for our methodological selection and serving as proof of concept for the proposed framework, with a clear explanation of how the performance metrics compare to those in state-of-the-art works, as follows:

"Ablation study

The ablation study provides evidence in support of the justification for the key components of AMD-FV. Five models are evaluated, using the architecture of a 68 layered DPN+ neural network. Their summary descriptions are shown in Table 3.

The models were analyzed according to several performance metrics, such as model complexity (i.e., parameter number and model size), computational time measured in FLOPs, inference time in seconds, and accuracy rate. Note that model complexity, computational time, and inference time are closely related to the computational power of the machine, whereas accuracy rate is related to model performance on the input data. Tables 4 and 5 illustrate the computational complexity and inference times of the evaluated models, respectively. It should be noted that the same CNN architecture was used in all of the models. Therefore, the chosen metrics will have similar values, providing the means to perform a fair comparison of impact of loss functions on the basis of the accuracy rate.

Robustness of DPN+

In this sub-section, we focus on the analysis of the proposed Dual Path Network+ over the Dual Path Network, so as to provide a fair comparison. Both DPN and DPN+ were trained on the same

dataset (MS1Mv2 [10]), under the same settings (See Section for details) and loss function (i.e., CosFace loss [9]). The models were then evaluated on the five benchmark face datasets. The obtained results support the claim that the proposed DPN+ model (DPN+68-C) extracts more discriminative features than those of the DPN model (DPN68-C). The verification accuracy of DPN+68-D is 0.17%, 1.78% and 2.94% higher than that of DPN68-C on the LFW, CPLFW and Megaface datasets, respectively (Table 9). A slight drop of 0.07% in the performance of the DPN+68-C compared to DPN68-C is observed in the case of the CALFW dataset. Finally, the ROC curves in Fig. 3 demonstrate that the DPN+68-C model significantly surpasses the DPN68-C model on the IJB-B dataset.

Robustness of AMD-FV

Next, we provide a comparison between the most relevant state-of-the-art angular loss function models, and the proposed AMD-FV model. The SphereFace model [8] proposes a multiplicative angular margin, where the margin value is multiplied by the angle, which, in turn, provides for a fixed distance between the samples. The multiplicative property of the margin highly influences the angle. When the angle decreases towards zero, the margin vanishes (see Fig. 6(a)). The CosFace [9] and ArcFace models [10] introduce the additive angular margin. In CosFace model [9], the margin value is subtracted from the cosine value (Eq. 2), which overcomes the limitation of the vanishing margin (see Fig. 6(b)). Compared with the CosFace model [9], the ArcFace model [10] adds a margin to the angle. The ArcFace model [10] also has a vanishing margin problem. However, because of the additive property, a fixed margin value does not significantly affect the angle (see Fig. 6(c)). In the proposed model, AML automatically estimates the margin using the input data, so that it does not vanish. Specifically, it uses the cross product of the feature map to estimate the margin according to Eq. 3 and subtracts the margin from the cosine value (Eq. 5) (see Fig. 6(d)). By adaptive estimation of the margin and the scale hyperparameters, AML provides an alternative to the otherwise, time-consuming training of the trial and error approach.

The principal objective in this research is the development of an automated procedure for the selection of the margin and scale hyperparameters, which resulted to the proposed AML function. Analysis of the experimental results demonstrates that the accuracy obtained by the AMD-FV model surpasses the performance of both state-of-the-art AdaptiveFace [11] and Adacos models [12]. Note that the mean verification accuracy on the LFW dataset is almost saturated and thus, a small increase in the accuracy has a significant impact. The verification accuracy achieved by the AMD-FV model is 99.75%, which is higher than that of AdaptiveFace at 99.63% and Adacos at 99.71% (Table 6). In addition, the accuracy of the AMD-FV model is higher than that of the baseline model (DPN+68-A) on all the testing datasets. It is worth noting that the introduction of AML results to an average improvement of 2.338% compared to using CESL (Table 10 and Fig. 4). The verification accuracies obtained by the DPN+68-B model (DPN+ with ArcFace loss) and the DPN+68-D model are similar to one another (Table 8). Specifically, the use of ArcFace loss led to outperforing the DPN+68-D model by a margin of 0.19%, on average, suggesting that the proposed adaptive DPN+68-D model not only surpasses the state-of-the-art adaptive margin-based models, but also favorably compares with the manually optimized margin-based model (i.e., ArcFace). Fig. 5 provides a visual comparison of the computational complexity of the DPN+68-D model and the ArcFace model [10] (i.e., ResNet with 100 layers). It is shown that the proposed AMD-FV model leads to substantial reductions of 32%, 26.92% and 41.91% in terms of the number of layers, parameters and FLOPS, respectively.

Comparison with state-of-the-art methods

In this sub-section, we consider the performance of AMD-FV in regards to related state-of-the-art approaches. The mean verification accuracies acquired on the LFW dataset are presented in Table 11. The proposed AMD-FV method attains a competitive mean verification accuracy of 99.83%, surpassing the performance of the majority of previously published methods e.g., [42], [8], [43], and [44]. Arcface [9] exhibits a slightly higher accuracy compared to AMD-FV by 0.08%. However, in comparison to the manually optimized CosFace model [9], the proposed AMD-FV method with automated hyperparameter selection demonstrates an improvement of 0.02%. This suggests that the automated hyperparameter selection process of AMD-FV could serve as an alternative to the empirically determined hyperparameter values of the CosFace model, eliminating the need for time-consuming trial and error. Furthermore, in comparison to the other two adaptive models, namely AdaptiveFace [11] and Adacos [12], AMD-FV demonstrates a marginal improvement of 0.13% and 0.04%, respectively.

IJB-B is considered to be a challenging dataset concerning occlusion, poses, and illumination. In Table 12, the True Acceptance Rate (TAR) is presented concerning various False Acceptance Rates (FAR) on the IJB-B dataset. The proposed AMD-FV model outperforms all other models. Specifically, it improves on the VGGFace2 model by 6%, and by 4.6% and 3.9% compared to MN-V and MN-VC, respectively, at a FAR of 0.001. At a FAR of 0.01, the proposed AMD-FV surpasses VGGFace2, MN-V, and MN-VC models by 2.83%, 2.23%, and 1.93%, respectively. At a FAR of 0.1, AMD-FV's performance marginally outperforms that of the VGGFace2, MN-V, and MN-VC. This insight underscores the improved performance of AMD-FV compared to state-of-the-art methods on the IJB-B dataset.

The Rank-1 identification scores of existing methods on the Megaface dataset are presented in Table 13. The gallery set of 1M distractor images in Megaface presents a wide range of poses, ages, and illumination changes, making this dataset exceptionally challenging for face verification. Notably, the proposed AMD-FV method outperforms several state-of-the-art models, such as the CosFace model [9], Sphereface model [8], and FaceNet [42], with considerable margins (i.e., greater than 10%). However, when compared to the adaptive hyperparameter estimation models of AdaptiveFace [11] and Adacos [12], the results of AMD-FV are lower. This difference is attributed to Megaface's use of a large number of distractor images in its verification protocol, which affects AMD-FV's margin hyperparameter estimation values computed based on the mean. It is worth noting that the primary objective of this work is to adaptively estimate the large margin cosine loss of CosFace [9]. In this regard, the proposed AMD-FV model achieves a Rank-1 score of 92.16%, significantly surpassing CosFace by 9.44%.

The mean verification accuracy on the Cross Age LFW (CALFW) [39] and Cross Pose LFW (CPLFW) datasets [40] is presented in Table 14. These datasets extend the LFW dataset by introducing variations in age and pose, thereby increasing the complexity level. On the CALFW dataset, AMD-FV significantly outperforms the Center loss [47], SphereFace [8], and VGGFace2 models [5] by 10%, 5.21%, and 4.94%, respectively. Similarly, on the CPLFW dataset, the mean verification accuracy of AMD-FV surpasses that of the Center loss [47], SphereFace [8], and VGGFace2 models [5] by 13.52%, 9.60%, and 7.00%, respectively."

Moreover, a number of new Tables and Figures were included to support these additional results and discussions. In the revised manuscript, the following new Tables were included:

Table 3: overview of the experimental models used in the ablation study

Table 4: performance comparison of the models w.r.t computational parameters

Table 5: performance comparison w.r.t inference times

Table 7: comparison of mean verification rates between AMD-FV and ArcFace loss

Table 8: comparison of MVA, precision, and recall with ArcFace loss

Table 9: comparison between DPN and DPN+ using CosFace loss

Table 10: comparison bebetween AMD-FV and baseline model

Moreover, the following new Figures were included:

Figure 3: ROC comparison between DPN and DPN+ using CosFace loss

Figure 4: ROC comparison between AMD-FV and baseline model

Figure 5: Computational parameters comparison between ArcFace and AMD-FV

Figure 6: Geometric representation of angular loss functions

R2.C5- Where is the ablation study. Add this subsection to your experimental results sections.

Response: Thank you for this comment, which was comprehensively addressed under R2.C4.

R2.C6- There are no mention of the metrics equations, and how did you verify your results with these metrics.

Response: Thank you for this recommendation, which was addressed as follows. First, we included the explanation and definition of all metrics in the revised results section as follows:

"*The performance metrics used in the subsequent sub-sections are as follows:*

True Positive Rate (TPR): TPR is defined as the ratio of samples identified as true positives (TP) over the total number of positives (P):

TPR = TP/P

The total number of samples identified as positive is given by:

P = TP+FN

where FN relates to the false negatives.

False Positive Rate (FPR): FPR is defined as the ratio of samples identified as false positives (FP) over the total number of positives (P):

FPR = F P/P

Mean Verification Accuracy (Accuracy): This metric measures the overall correctness of the model in verifying identities. It is defined as the ratio of correctly predicted instances to the total instances:

Accuracy = (TP + TN)/(TP + TN + FP + FN)

Precision: Precision quantifies the accuracy of the positive predictions made by the model. It is calculated as the ratio of true positives to the sum of true positives and false positives:

Precision = T P/(TP+FP)

Recall: Recall measures the model's ability to identify all relevant instances. It is defined as the ratio of true positives to the sum of true positives and false negatives:

Recall = TP/(TP+FN)

True Acceptance Rate (TAR): TAR is measured as the ratio of the number of positive matches, Np, over the sum of the number of positive matches and the number of false rejects, Nr :

TAR = Np/(Np+Nr)

False Acceptance Rate (FAR): A complimentary metric to TAR, FAR relates to the proportion of false accepts, i.e., FP , which are incorrectly accepted as genuine attempts, over the total number of impostor attempts, i.e., the sum of FP and TN :

FAR = FP/(FP+TN)

Rank-1 score: This metric provides a measure of the FV system's ability to correctly identify the top match for a given query image from a set of images. It is defined as the proportion of queries where the correct match appears as the top-ranked candidate in the system's output:

Rank-1 = M/Q

where M and Q relate to the number of queries where the true match ranks as top, and the total number of queries, respectively."

In regards to the results verification, these were applied across a diverse number of datasets, so as to confirm the performance of the proposed AMD-FV. Moreover, a substantial comparison study with state-of-the-art approaches using the same set of metrics is provided in the sub-section Comparison with state-of-the-art methods, please see the penultimate paragraph of our response to comment R2.C4.
