# Supplementary material for: AMD-FV: Adaptive margin loss and dual path network+ for deep face verification
Source: PLoS One. 2025 May 28;20(5):e0324485. doi: 10.1371/journal.pone.0324485 (PMC12118897; doi:10.1371/journal.pone.0324485)
Supplement: Attachment — Submitted filename: PONE-D-24-28461R2- Response to the Reviewers comments.pdf [file pone.0324485.s003.pdf]

Reviewer #1:

(No Response)

Reviewer #3:

We would like to thank the reviewer for the rigorous review of our work and their constructive comments, which have led to substantial improvement in the presentation and communication of the theory and results presented in this work. In what follows we provide a point-by-point response to all comments raised by the reviewer.

R3.C1 - No need to recite the models every time they are mentioned.

Response: Thank you for your valuable feedback. We revised the manuscript to eliminate redundant model recitations by consistently using abbreviated notations. These edits streamline the narrative and improve readability.

R3.C2 - It is unclear how Algorithm 1 obtains the values of N and R used in Eq. (4). Please add these variables to the algorithm's requirements.

Response: Thank you for highlighting this ambiguity. We updated Algorithm 1 to explicitly define **N** (number of training classes) and **R** (maximum adaptive range of AML, derived from Appendix B) as inputs to the scale hyperparameter computation in Step 5. These variables are now clearly stated in the algorithm description.

R3.C3 - The name of the DPN model with CosFace is listed as DPN68-E in Table 3 and DPN68-C in Table 9. Please ensure consistency.

Response: Thank you for identifying this inconsistency. We rectified this error by updating the model name in Table 9 from "DPN68-C" to "DPN68-E" to align with the nomenclature in Table 3. The revised manuscript now uniformly references the DPN with CosFace loss as "DPN68-E" across all sections, tables, and analyses. We also performed a thorough cross-check to ensure such discrepancies do not persist elsewhere in the document.

R3.C4 - Please provide one or two sentences to justify the performance drop of DPN+68-C on the CALFW dataset.

Response: Thank you for raising this important point. The marginal performance drop of DPN+68-C on CALFW stems from its training focus on pose invariance and real-world robustness, rather than explicit age-invariant modeling. While current leading methods may employ synthetic aging augmentation, our

framework prioritizes practical deployment scenarios where pose variations and occlusions are more prevalent than extreme age gaps. We explicitly addressed this in the revised "Robustness of DPN+" section, clarifying its implications and future mitigation strategies, as follows:

“The slight drop in performance of the DPN+68-C model in Table 9 on the CALFW dataset occurs because the model has not been specifically trained to handle age gaps in photos of the same person. While it excels at matching faces with varying angles (CPLFW), crowded scenes (MegaFace), and general recognition (LFW), aging variations, such as wrinkles, facial shape-shifts are distinct and unique challenges. To address this, state-of-the-art approaches employ dedicated techniques, e.g., synthetically "aged" face examples during training, which the proposed model does not provide for. However, DPN+’s stronger performance on pose variations and complex real-world scenarios suggests it is better suited for practical applications, where camera angles and crowded environments matter more than extreme age differences. To address aging variations within the current framework, it is possible to augment the training dataset with aging-specific instances or perform fine-tuning for aging effects, without compromising on the model’s current strengths.”

R3.C5 - The results of Table 7 are not discussed in the manuscript.

Response: Thank you for highlighting this oversight. We revised the “Robustness of AMD-FV” section to include a detailed analysis of Table 7, which compares the performance of DPN+68-B and DPN+68-D models as follows:

“Moreover, Table 7 compares the performance of two DPN+ architectures, i.e., DPN+68-B (trained with ArcFace loss) and DPN+68-D (trained with AML loss). The results reveal a marginal performance differential between the two models. DPN+68-D exhibits near-equivalent accuracy to DPN+68-B, with minor deviations, i.e., -0.03% on LFW, -0.19% on CALFW, and -0.35% on CPLFW. Notably, DPN+68-D outperforms DPN+68-B by +0.28% on the IJB-B benchmark.”

R3.C6 - It appears that the ArcFace loss function outperforms the proposed AML in the results shown in Tables 7, 8, 11, and 13. Although there is a brief mention at the end of the “Robustness of AMD-FV” section stating that AML is less complex, I believe further discussion is needed at the beginning of this section.

Response: We would like to thank the reviewer for raising this comment, which provides us with the opportunity to clarify the comparison between ArcFace loss and AMD-FV. We agree with the reviewer that Tables 7, 8, 11, and 13 demonstrate that ArcFace achieves marginally superior performance on certain benchmarks, however, this apparent advantage stems from ArcFace’s reliance on manually optimized hyperparameters, performed on each of the employed datasets. In contrast, AML eliminates the need for manual tuning by automatically adapting the margin and scale parameters based on input dissimilarity and class distribution. This automation inherently introduces a trade-off as follows:

Manual Tuning vs. Automation: ArcFace’s manual margin selection allows fine-grained optimization for benchmark metrics, however, it requires extensive trial-and-error, which is impractical for large-scale,

real-world deployment. Instead, AML replaces this process with data-driven adaptation, sacrificing marginal accuracy gains for generalizability and scalability.

Computational Efficiency: As highlighted in Fig. 5, AML respectively reduces model complexity by 32%, 26.9% and 41.9% in terms of the number of layers, parameters, and FLOPs compared to ArcFace. This efficiency makes AML more suitable for resource-constrained environments.

Performance Contextualization: The slight performance gap is negligible in practical applications, however, it underscores the cost of automation. AML avoids the laborious hyperparameter search, making it a preferable choice for dynamic datasets, where manual tuning is infeasible.

To address the reviewer's comment, the following discussion has been included in the manuscript:

"*Tables 7, 8, 11, and 13 demonstrate that ArcFace achieves marginally superior performance on certain benchmarks, however, this apparent advantage stems from ArcFace's reliance on manually optimized hyperparameters, performed on each of the employed datasets. In contrast, AML eliminates the need for manual tuning by automatically adapting the margin and scale parameters based on input dissimilarity and class distribution. This automation inherently introduces a trade-off as follows:*

***Manual Tuning vs. Automation**: ArcFace's manual margin selection allows fine-grained optimization for benchmark metrics, however, it requires extensive trial-and-error, which is impractical for large-scale, real-world deployment. Instead, AML replaces this process with data-driven adaptation, sacrificing marginal accuracy gains for generalizability and scalability.*

***Computational Efficiency**: As highlighted in Fig. 5, AML respectively reduces model complexity by 32%, 26.9% and 41.9% in terms of the number of layers, parameters, and FLOPs compared to ArcFace. This efficiency makes AML more suitable for resource-constrained environments.*

***Performance Contextualization**: The slight performance gap is negligible in practical applications, however, it underscores the cost of automation. AML avoids the laborious hyperparameter search, making it a preferable choice for dynamic datasets, where manual tuning is infeasible.*"

R3.C7 - ArcFace has the best result in Table 13, not Adacos.

Response: Thank you for spotting this oversight. We updated Table 13 to accurately reflect ArcFace as the top-performing method, as noted in the comment. The ArcFace results are now shown in bold in the revised table to emphasize their superior performance and improve clarity.

R3.C8 - Closing parentheses is missing in table 5. "Inference Time (s)"

Response: Thank you for identifying the formatting oversight in Table 5. The missing closing parenthesis in the "Inference Time (s)" column header has been added, and the table has been revised accordingly.

R3.C9 - It should be Fig. 5 instead of Fig. 6 in the last paragraph of the "Discussion" section. The sentence should read: "Fig. 5 provides a visual comparison …"

Response: We are thankful to the reviewer for identyfying this discrepancy. We have the figure reference to **Fig. 5** in the final sentence of the "Discussion" section.

Reviewer #4:

We would like to thank the reviewer for the rigorous review of our work and their constructive comments, which have led to substantial improvement in the presentation and communication of the theory and results presented in this work. In what follows we provide a point-by-point response to all comments raised by the reviewer.

R4.C1 - the experimental setup section could be more detailed, including specific training parameters, optimiser settings, data preprocessing steps, etc.

Response: To respond to the reviewer's comment the following revisions were made to enhance methodological clarity and reproducibility. The revised section explicitly outlines the training parameters, optimization strategies, data preprocessing steps, and evaluation protocols as follows:

"Training Configuration: *The experiments were conducted on a hardware setup of three NVIDIA TITAN X GPUs interconnected via NVLink, with a global batch size of 128 (distributed as 42–43 samples per GPU). Synchronized batch normalization was used to ensure consistent gradient updates across GPUs. The learning rate began at 0.1 with a 5-epoch linear warmup phase, followed by a cosine annealing schedule which gradually reduced the rate to a minimum of 1e-6. Stochastic Gradient Descent (SGD) with momentum (μ = 0.9) was employed as the optimizer, coupled with gradient clipping to a maximum L2 norm of 5.0 to prevent exploding gradients. The regularization strategy included weight decay (5e-4 applied exclusively to the convolutional and fully connected layers), label smoothing (ε = 0.1), and stochastic depth with a DropPath probability of 0.2.*

Data Preprocessing Pipeline: *Face detection was performed using MTCNN with 5-landmark localization (eyes, nose, and mouth corners). Alignment involved a similarity transformation based on eye coordinates, supplemented by 3D face reconstruction for images with extreme poses (yaw or pitch exceeding 45°). Augmentation strategies included random horizontal flipping (50% probability), color jitter (brightness ±0.2, contrast ±0.15), and patch masking (up to 15% of the image area). Input normalization comprised channel conversion, per-channel mean subtraction ([91.4953, 103.8827, 131.0912]), and 8-bit quantization with histogram equalization.*

Evaluation Protocols: *For standard benchmarks such as LFW, CALFW, and CPLFW, we adopted a 10-fold cross-validation protocol with unrestricted settings. MegaFace evaluation followed the "Large" protocol (1 million distractor images), while IJB-B verification metrics (TAR@FAR) were computed over 10,000 genuine and 8 million impostor pairs. All face embeddings were compared using cosine similarity, with decision thresholds optimized via the ROC convex hull method.*

Computational Environment: *The implementation used PyTorch 1.12.1 with CUDA 11.6, leveraging mixed FP16/FP32 precision through NVIDIA Apex AMP. Parallelization was managed via PyTorch's DataParallel module with NCCL backend, and all random processes were fixed to a seed value of 42 for reproducibility purposes."*

R4.C2 - despite the ablation study, the specific contribution of each of the AML and DPN+ components to the performance improvement of the final results is not detailed.

Response: Thank you for raising this crucial point. To clarify the individual contributions of AML and DPN+, we expanded the analysis in "Discussion" section as follows:

"***DPN+ Contribution***
Table 9 demonstrates that DPN+68-C (with CosFace loss) outperforms DPN68-E (original DPN with CosFace) by 0.17% on LFW and 1.78% on CPLFW. This improvement stems directly from DPN+'s redesigned Block-1, which preserves low-level spatial features through three sequential 3x3 convolutions (vs. DPN's 3x3 maxpool with stride=2). The absence of downsampling in Block-1 reduces information loss, particularly benefiting pose-sensitive datasets like CPLFW.

AML Contribution
Table 7 reveals that AML (DPN+68-D) achieves comparable accuracy to ArcFace (DPN+68-B) despite the use of automated hyperparameter selection (i.e., 99.75% vs. 99.78%) on LFW. The marginal performance drop of 0.03% reflects the cost of replacing manual tuning with AML's data-driven adaptation. However, AML reduces training complexity by eliminating tedious and laborious effort on manual hyperparameter search.

Integration of DPN+ with AML
The combined AMD-FV model (DPN+68-D) achieves 41.9% lower FLOPs than ArcFace (Fig. 5) by combining AML's efficient scaling with DPN+'s parameter reduction."

R4.C3 - although a method for automatic selection of hyperparameters is proposed, the specific range of values and tuning strategies for these hyperparameters are not detailed.

Response: Thank you for highlighting this critical aspect of our work. The proposed framework alleviates the burden of manual hyperparameter tuning by algorithmically deriving optimal values during training, as outlined in Appendix B. To ensure clarity, we summarize the key mechanisms below:

Margin hyperparameter

While Eq. 3 calculates margins as $m = \frac{1}{n}\sum\left|W_j^T \cdot x_i\right|$, practical implementation requires

bounding m∈[0,2]. This stems from the trigonometric identity cosθj−sinθj (Appendix B), where theoretical analysis shows $\sqrt{1-\left(W_j^T \cdot x_i\right)^2}$ cannot exceed 1 for normalized features, yielding m≤2.

Scale hyperparameter

The scale formula $s = log(N) \cdot e^{\sqrt{2}}$ (with the range $R = \sqrt{2}$) is designed to mitigate class imbalance in large datasets (MS1Mv2's 85K classes) via log(N), aligning with standard practices for high-cardinality classification, and amplifies small angular differences using $e^{\sqrt{2}}$, thus preventing gradient saturation in the softmax function.

R4.C4 - The methodology section needs to detail the specific implementation and optimisation strategy of each component to ensure that the proposed methodology can be clearly understood and reproduced by the reader.

Response: Thank you for this comment. The revised methodology section explicitly outlines the training parameters, optimization strategies, data preprocessing steps, and evaluation protocols. Please see our response to comment R4.C1 of this reviewer.
